# Investigating Changes in Cardiac Function and Structure of Left Ventricle by Speckle-Tracking Echocardiography in Patients With Hyperthyroidism and Graves' Disease

**DOI:** 10.3389/fcvm.2021.695736

**Published:** 2021-10-27

**Authors:** Binyi Li, Zheng Li, Yong Huang

**Affiliations:** ^1^Department of Ultrasound, The People's Hospital of Danyang, Danyang Hospital of Nantong University, Danyang, China; ^2^Department of Endocrinology, The People's Hospital of Danyang, Danyang Hospital of Nantong University, Danyang, China

**Keywords:** left ventricle, speckle-tracking echocardiography, hyperthyroidism, Graves' disease, meta-analysis

## Abstract

Subclinical hyperthyroidism is a common thyroid dysfunction that can lead to cardiovascular complications. It is necessary to understand the treatment strategy in clinical practice. This study aimed to investigate the changes in cardiac function and left ventricular (LV) structure by speckle-tracking echocardiography in patients with hyperthyroidism and Graves' disease. PubMed, Medline, Cochrane Library, Embase, and SinoMed were searched for targeted articles, from inception till November 26, 2020, without any language restriction. All studies that evaluated changes in cardiac function and LV structure by speckle-tracking echocardiography in patients with hyperthyroidism and Graves' disease were screened. Included studies met the following inclusion criteria: (1) study population diagnosed with hyperthyroidism or Graves' disease; (2) patients without treatment and are undergoing speckle-tracking echocardiography; (3) necessary data could be extracted from original studies; (4) studies published in English or Chinese; and (5) if the study population was duplicated, only one study from the same institution that provided detailed information or newly published article was selected. All relevant articles from the above databases were screened and assessed according to the inclusion criteria by two reviews independently. Inverse variance methods with random-effects were employed to pool the mean differences (MDs) and the corresponding 95% confidence intervals (CIs). Ten studies with 483 patients and 434 healthy controls were included for data extraction and meta-analysis. On comparing patients with healthy controls, two-dimensional echocardiography revealed significant differences in several parameters including interventricular septal thickness (IVST) [mean difference (MD): 0.43, 95% CI = 0.12–0.73, *P* < 0.05] and left ventricular end systolic diameter (LVESD) (MD: 1.42, 95% CI = 0.33–2.52, *P* < 0.05). Moreover, there were significant differences in left ventricular ejection fraction (LVEF) (*P* < 0.05), global longitudinal strain (*P* < 0.05), and global circumferential strain (*P* < 0.05) demonstrated by three-dimensional echocardiography. These findings suggested that left ventricle (LV) function evaluated by speckle-tracking echocardiography showed significant impairment in patients with hyperthyroidism. However, additional original studies and meta-analyses are warranted for an in-depth investigation.

## Introduction

Subclinical hyperthyroidism is a common thyroid dysfunction that leads to cardiovascular complications such as left ventricular (LV) diastolic dysfunction, which is characterized by increased LV mass and a high risk of supraventricular arrhythmias (1–3). The cardiovascular system is affected by hyperthyroidism in several ways, causing various changes including increased heart rate and contractility, reduced systemic vascular resistance, increased preload, and decreased afterload (4, 5). Thyroid hormones could influence the structure of LV due to long-term hyperthyroidism, leading to concentric cardiac hypertrophy (6, 7). If left untreated for a long time, this could lead to heart failure (8, 9).

With the image-processing algorithm for the two-dimensional digital echocardiography, small and stable myocardial footprints were generated through the ultrasound-myocardial tissue interactions within defined regions of interest (10). Speckle-tracking echocardiography is a newer technique that provides a more detailed image of structural and functional changes that occur during excessive thyroxine release and has been widely used in cardiology practice (10). Speckle-tracking echocardiography is independent of the ultrasound beam angulation and has low intra- and inter-operator variability.

Several recent studies (6, 11, 12) have reported the results of LV changes using speckle-tracking echocardiography. However, the role of speckle-tracking echocardiography in detecting LV dysfunction has not been elucidated yet, although it has clinical relevance in clarifying the pathophysiology of LV changes and the development of heart failure in subjects. Previously published studies on this topic provide evidence for conducting a powerful and persuasive systematic review and meta-analysis. Hence, this study systematically evaluated the changes in cardiac function and LV structure in hyperthyroidism with Graves' disease by speckle-tracking echocardiography.

## Methods

### Literature Search

PubMed, Medline, Cochrane Library, Embase, and SinoMed databases were searched for targeted studies, from their inception till November 26, 2020, without any language restriction. The individual and joint keywords used to search potential literature were as follows: “Hyperthyroidism,” “Graves' disease,” “speckle tracking,” “speckle-tracking,” and “echocardiography.” The search terms considered were as broad as possible to obtain relevant studies. Moreover, the bibliographies of all relevant studies and reviews were reviewed for any additional eligible studies. Google Scholar was also searched for relevant articles. The current study was conducted according to the guidelines of the Preferred Reporting Items for Systematic Reviews and Meta-Analyses (PRISMA) statement (13).

### Eligibility Criteria

The inclusion criteria were as follows: (1) study population diagnosed with hyperthyroidism or Graves' disease; (2) patients without treatment who are undergoing speckle-tracking echocardiography; (3) necessary data could be extracted from original studies; (4) studies published in English or Chinese; and (5) if the study population was duplicated, only one study from the same institution that provided detailed information or newly published article was selected.

Case reports, letters, reviews, commentaries, conference abstracts, animal model studies or *in vitro* experiments, studies in languages other than English and Chinese, and studies with unavailable data were excluded.

### Data Extraction

All relevant articles from the above databases were screened and assessed according to the inclusion criteria by two reviews independently. All necessary information of the standard-compliant studies was extracted using a standardized form by the above two authors independently, and a consensus was reached on all items after discussion with a third reviewer. The following information was extracted for all included studies: study characteristics (first author, year of publication, country, sample size, and study design), patient and/or control characteristics (e.g., mean age and female percentage), and results of speckle-tracking echocardiography.

### Quality Scoring of Studies

Newcastle–Ottawa Scale (NOS), a quality assessment and validity tool that independently assesses the methodological quality for meta-analysis of observational studies (14), was utilized to assess the quality of the included studies by two reviewers independently.

Newcastle–Ottawa Scale provided grades for meta-analysis of observational studies based on three categories: selection, comparability, and exposure. If the cases were defined adequately, the detailed criteria for the three assessments included the representativeness of the cases, the process of selection and definition for controls, comparability of cases and controls based on design or analysis, ascertainment of exposure, the same method of ascertainment for cases and controls, and nonresponse rate. A study could be awarded a maximum of one star for each numbered item within the selection and exposure categories, and a maximum of two stars could be given for comparability. The score ranged from 2 to 9 points. A scale of 0–2 points indicated poor quality, 3–5 points indicated medium quality, and 6–9 points indicated high quality. To explore potential heterogeneity, studies with lower or medium quality were used for sensitivity analysis.

### Statistical Analysis

Inverse variance methods with random-effects were employed to pool the mean differences (MDs) and the corresponding 95% confidence intervals (CIs). The heterogeneity based on *I*^2^ statistics was used to assess the consistency of the effect sizes. Heterogeneity was categorized into with and without significant heterogeneities according to the value of *I*^2^ ≥ 50% and <50% (15), respectively. To explore the sources of heterogeneity, individual studies were sequentially excluded from demonstrating the overall impact of all included studies where *I*^2^ ≥ 50% (significant heterogeneity). Publication bias was assessed by Begg's rank correlation (16) and Egger's weighted regression methods (17). Statistical analyses and Begg's and Egger's tests were performed by STATA 15.0 (Stata Corporation, College Station, TX, USA). A *P* < 0.05 was considered statistically significant.

## Results

### Study Selection

**Figure 1:**
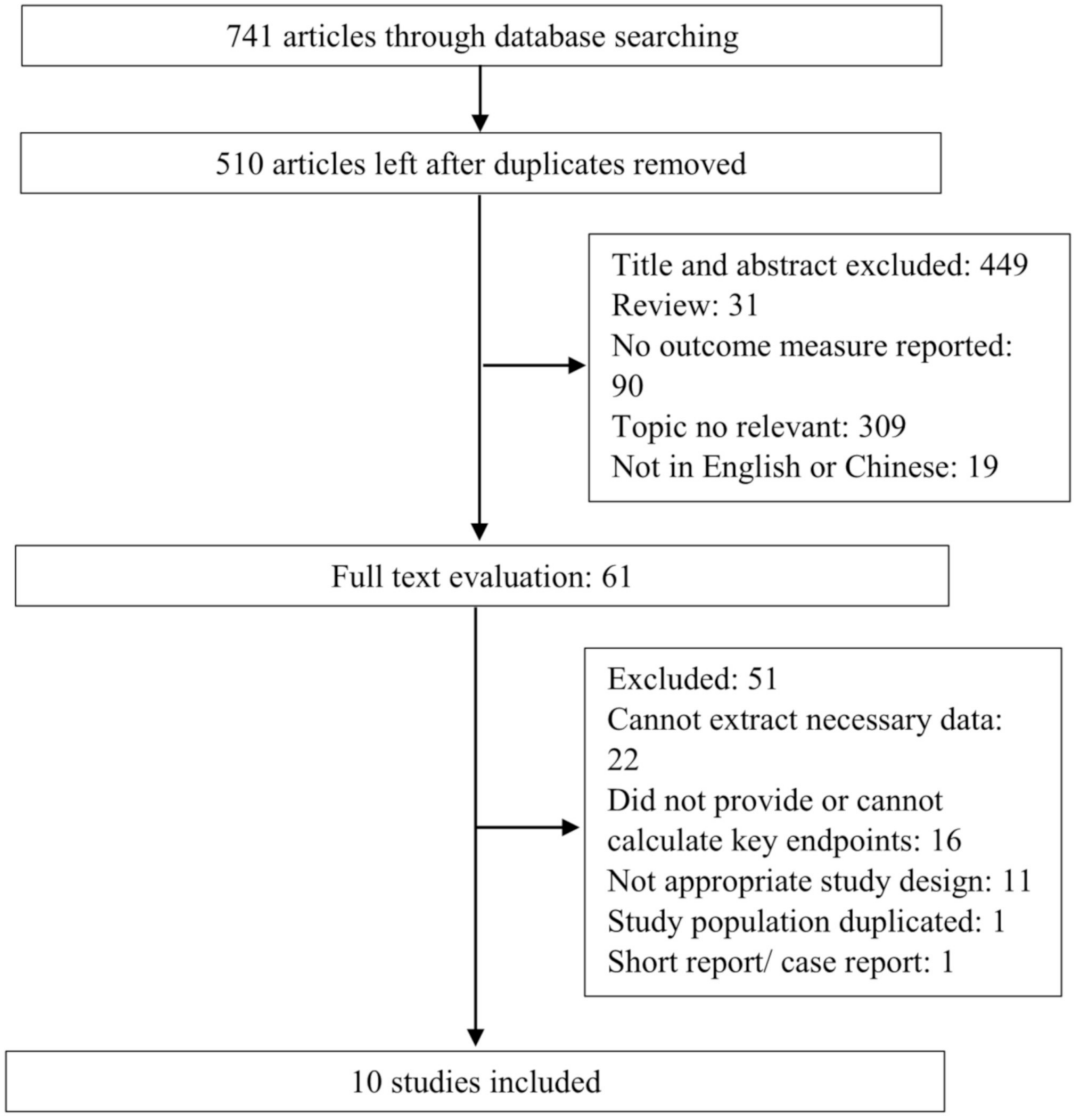
Flowchart of the study selection process.

The flowchart of the study selection process is shown in Figure 1. The systematic literature search yielded 741 studies, and 231 of these were excluded due to duplication. Based on the inclusion and exclusion criteria, 449 abstracts and titles were excluded. A total of 61 full-length manuscripts were retrieved, with 11 excluded due to inappropriate study design including enrolled cases with multiple comorbidities and cases receiving treatments. Ultimately, 10 articles (6, 11, 12, 18–24) were included for data extraction and meta-analysis.

### Study Characteristics

In total, 10 studies with 483 patients and 434 healthy controls were included. These studies were published between 2015 and 2020. All included studies adopted a case-control design and the majority of the selected controls were according to age and sex. The eligible studies were conducted in China (*n* = 5) (6, 18–20, 24), Italy (*n* = 3) (21–23), Greece (*n* = 1) (12), and Turkey (*n* = 1) (11). All studies enrolled healthy individuals as the control group. The characteristics of participants are summarized in Supplementary Table 1.

### Quality Assessment of Studies

Based on NOS, six studies were awarded 7 points and four studies were awarded 6 points. All included studies were of high quality, and the scores for each study are presented in Supplementary Table 2.

### Two-Dimensional Echocardiography

**Figure 2:**
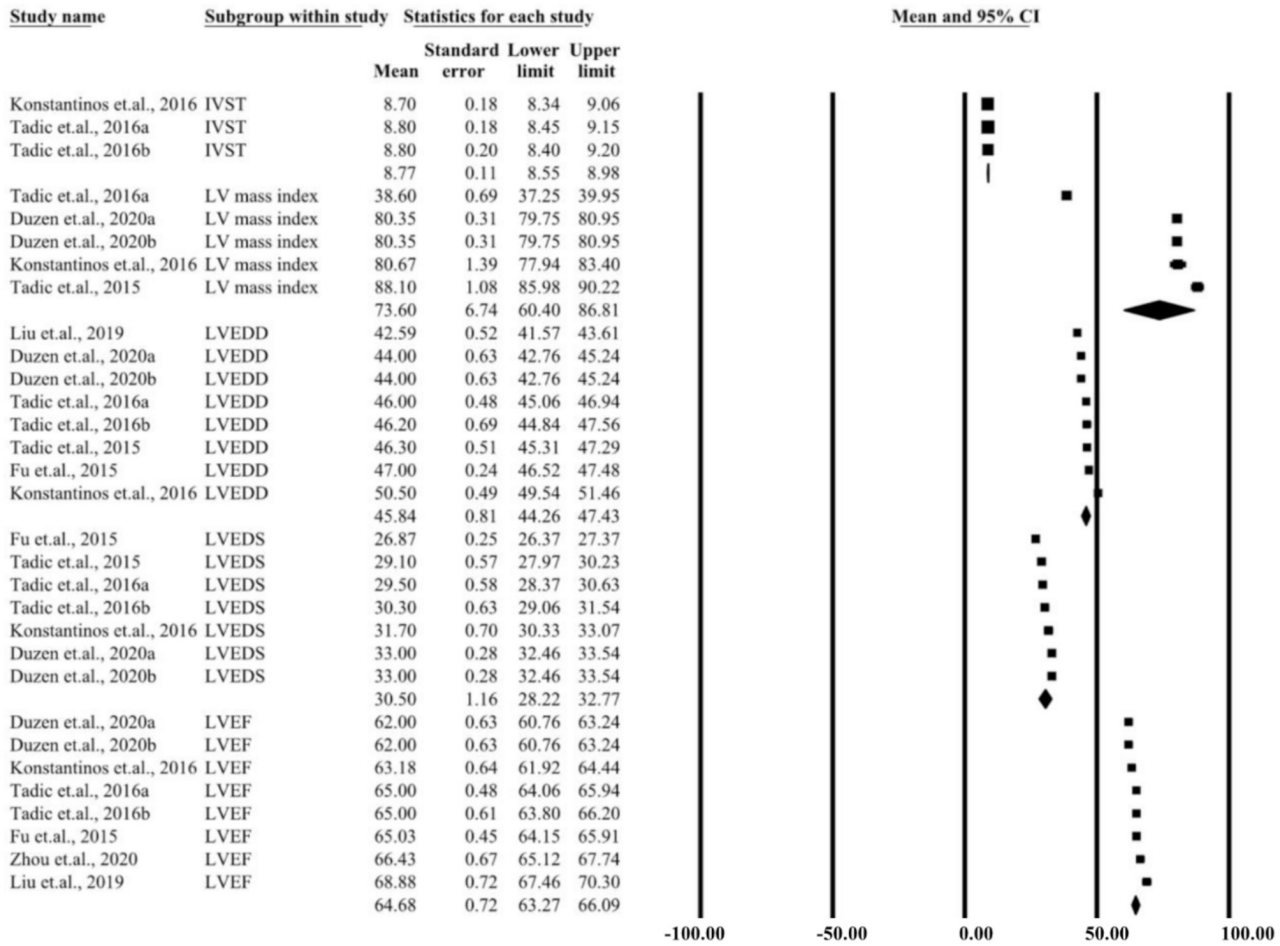
Summary of two-dimensional echocardiographic parameters for the left ventricular control group.

**Figure 3:**
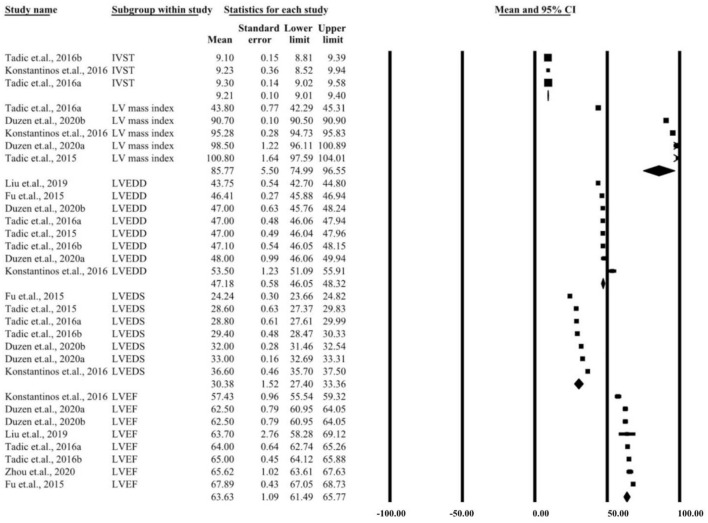
Summary of two-dimensional echocardiographic parameters for left ventricular case group.

Supplementary Table 3 shows the parameters of a single study by two-dimensional echocardiography. Nine studies included participants detected using two-dimensional echocardiography. Seven parameters, including left ventricular ejection fraction (LVEF), interventricular septal thickness (IVST), left ventricular end-diastolic diameter (LVEDD), left ventricular end systolic diameter (LVESD), and left ventricular mass index, were measured. The seven pooled parameters for patients and controls are presented in Figures 2, 3. When comparing the parameters between patients and controls, there were significant differences in IVST (MD: 0.43, 95% CI = 0.12–0.73, *P* < 0.05) and LVEDD (MD: 1.42, 95% CI = 0.33–2.52, *P* < 0.05). More details are presented in Supplementary Figure 1.

### Three-Dimensional Echocardiography

**Figure 4:**
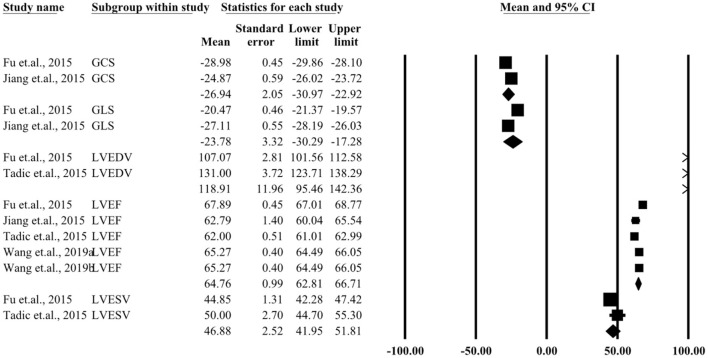
Summary of three-dimensional echocardiographic parameters for the left ventricular control group.

**Figure 5:**
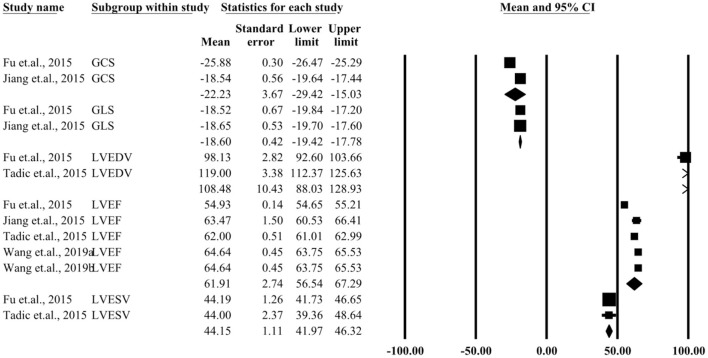
Summary of three-dimensional echocardiographic parameters for left ventricular case group.

Supplementary Tables 4–7 present several parameters of a single study by three-dimensional echocardiography. Five studies included participants detected by two-dimensional echocardiography. The 20 parameters that were measured included LVEF, LVEDD, LVESD, global longitudinal strain (GLS), global circumferential strain (GCS), global area strain (GAS), and global radial strain (GRS). The pooled parameters for patients and controls are presented in Figures 4, 5. Similar three-dimensional echocardiography results are shown in Supplementary Figure 2, in which the parameters such as LVEF (*P* < 0.05), GLS (*P* < 0.05), and GCS (*P* < 0.05) showed significant differences between the patient and the control group.

### Publication Bias

No potential publication bias was detected among the included studies according to Begg's rank correlation and Egger's weighted regression analyses (all *P* > 0.05, Supplementary Table 8).

## Discussion

To our knowledge, this was the first meta-analysis that focused on LV quantitative changes by speckle-tracking echocardiography in patients with hyperthyroidism and Graves' disease. A total of 10 studies with 483 patients and 434 healthy controls were included for data extraction and meta-analysis. All included studies were of high quality. Nine studies used two-dimensional echocardiography, while three-dimensional echocardiography was applied in five studies. The pooled MDs of IVST and LVEDD were shown to be significantly different by two-dimensional echocardiography between the patient and the control group. Similarly, on comparing the parameters for three-dimensional echocardiography between the two groups, LVEF (*P* < 0.05), GLS (*P* < 0.05), and GCS (*P* < 0.05) were found to be significantly different.

The majority of the included studies on echocardiography reported increased contractility and ejection fraction in the left ventricle in patients with hyperthyroidism and Graves' disease. A possible explanation for this might be that patients with severe hyperthyroidism could develop “high-output heart failure,” which could be related to tachycardia-mediated cardiomyopathy. In patients with long-term exogenous subclinical hyperthyroidism and preserved LVEF, speckle-tracking echocardiography analysis demonstrated the presence of impaired LV myocardial deformation (25). Therefore, for those patients, excess thyroid hormone could lead to deleterious effects on myocardial functioning, which is reversible upon restoration of thyroid status. However, whether these changes would be reversed after heart failure, were undetermined.

Long-term hyperthyroidism leads to systolic and diastolic dysfunction by affecting the heart rate, vascular resistance, oxidative stress, and renin-angiotensin system (26). Impaired systolic and diastolic functions were observed in the current study in patients with hyperthyroidism. Our findings showed that LVSF, as estimated by two- and three-dimensional echocardiography, did not differ from that of the healthy controls. The reason for the preserved LVSF, despite the decreased LVSD, could lie in the paradoxically elevated LV twist (23). LV twist involves the circumferential motion of the apex relative to the base of the heart, enabling the most effective contraction of the LV myocardial fibers, which could also be enhanced by overload conditions.

Although all included studies were of high quality, it is necessary to explore the limitations of the present meta-analysis when interpreting the results. First, all studies were case-control studies, and the nature of the study design could limit the ability to estimate causality and decrease the generalizability of the results. Second, half of the studies were conducted in China. These results could be affected by the environment, medical level, and genetic factors, which could only partially annotate the relationship, weakening the representativeness with regard to the target population. Moreover, due to the small size of study participants and various inclusion criteria, the heterogeneity across the studies was increased. Third, various ultrasound devices were used to assess the parameters of LV, and it might not be possible to ensure that all parameters were kept constant. This could be another cause of heterogeneity. Fourth, various studies have detected different parameters of LV, and only a few studies combined most of the pooled parameters, which also added to the heterogeneity. Finally, potential language bias could exist because our literature search only considered articles published in English or Chinese.

In conclusion, the current meta-analysis systematically assessed the changes of LV between patients and healthy controls by speckle-tracking echocardiography and evaluated several LV parameters that showed significant differences between the two groups. However, due to the limited number of included studies, original studies and meta-analyses with larger sample sizes from different counties are warranted to verify the current conclusions.

## Data Availability Statement

The original contributions presented in the study are included in the article/Supplementary Material, further inquiries can be directed to the corresponding authors.

## Author Contributions

BL carried out the studies, participated in collecting data, and drafted the manuscript. ZL performed the statistical analysis and participated in its design. YH helped to draft the manuscript. All authors have read and approved the final manuscript.

## Funding

This study was supported by the Zhenjiang Social Development Guidance Project (Grant Number FZ2015082) and the Danyang Social Development Science and Technology Support Project (Grant Number SF201511).

## Conflict of Interest

The authors declare that the research was conducted in the absence of any commercial or financial relationships that could be construed as a potential conflict of interest.

## Publisher's Note

All claims expressed in this article are solely those of the authors and do not necessarily represent those of their affiliated organizations, or those of the publisher, the editors and the reviewers. Any product that may be evaluated in this article, or claim that may be made by its manufacturer, is not guaranteed or endorsed by the publisher.

## Supplementary Material

Supplementary Figure 1 Mean differences in pooled two-dimensional echocardiographic parameters between patients and controls.

Supplementary Figure 2 Mean differences in pooled three-dimensional echocardiographic parameters between patients and controls.

Supplementary Table 1 Characteristics of study participants.

Supplementary Table 2 Quality assessment of included studies by Newcastle-Ottawa Scale.

Supplementary Table 3 Measurements of two-dimensional echocardiographic parameters in patient and control groups.

Supplementary Table 4 Measurements of three-dimensional echocardiographic parameters in patient group and the control group (part A).

Supplementary Table 5 Measurements of three-dimensional echocardiographic parameters in patient group and the control group (part B).

Supplementary Table 6 Measurements of three-dimensional echocardiographic parameters in patient group and the control group (part C).

Supplementary Table 7 Measurements of three-dimensional echocardiographic parameters in patient group and the control group (part D).

Supplementary Table 8 Publication bias of summarized outcomes.
