# Supplementary figures and images for: Investigating Changes in Cardiac Function and Structure of Left Ventricle by Speckle-Tracking Echocardiography in Patients With Hyperthyroidism and Graves' Disease
Source: Front Cardiovasc Med. 2021 Oct 27;8:695736. doi: 10.3389/fcvm.2021.695736 (PMC8578409; doi:10.3389/fcvm.2021.695736)

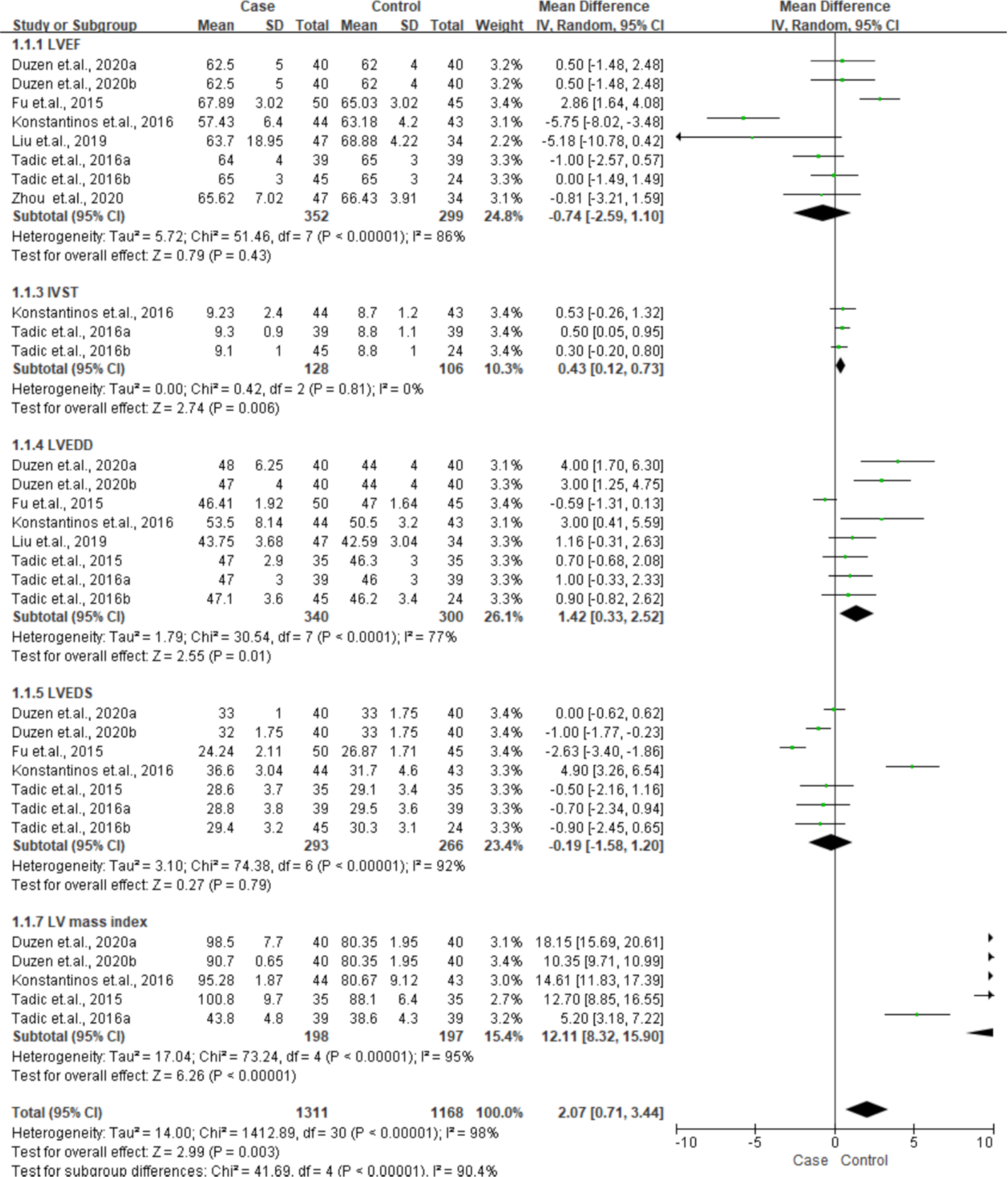

Supplement: Supplementary Figure 1 — Mean differences in pooled two-dimensional echocardiographic parameters between patients and controls. [file Image_1.TIF]

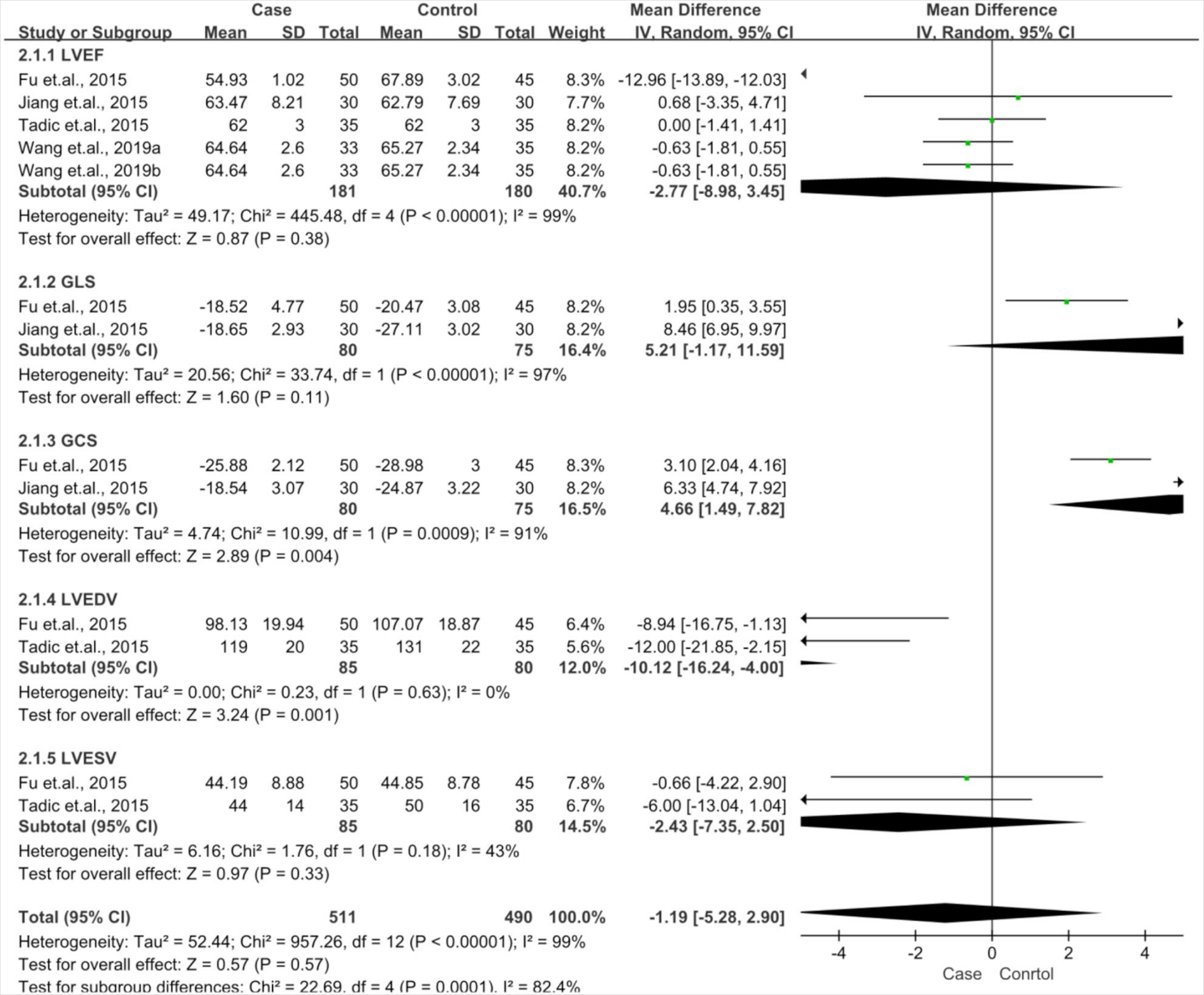

Supplement: Supplementary Figure 2 — Mean differences in pooled three-dimensional echocardiographic parameters between patients and controls. [file Image_2.TIF]
