# Supplementary material for: Investigating Changes in Cardiac Function and Structure of Left Ventricle by Speckle-Tracking Echocardiography in Patients With Hyperthyroidism and Graves' Disease
Source: Front Cardiovasc Med. 2021 Oct 27;8:695736. doi: 10.3389/fcvm.2021.695736 (PMC8578409; doi:10.3389/fcvm.2021.695736)
Supplement: Supplementary Table 1 — Characteristics of study participants. [file Table_1.DOCX]

**Supplementary Table 1. Characteristics of study participants**

| **Studies included** | **Country** | **Ultrasound tests (MHz)** | **Gender (male/female)** | | **Age (means ± SD)** | | **Sample size** | |
| --- | --- | --- | --- | --- | --- | --- | --- | --- |
|  |  |  | **Control** | **Patients** | **Control** | **Patients** | **Control** | **Patients** |
| Jiang et.al., 2015 | China | 1.7-3.3 | 6/24 | 7/23 | 31.21 ± 9.63 | 33.45 ± 8.24 | 30 | 30 |
| Fu et.al., 2015 | China | 1.9-3.8 | 15/30 | 15/30 | 35.60 ± 11.40 | 35.30 ± 7.70 | 50 | 45 |
| Tadic et.al., 2015 | Italy | 2.5 | 2/41 | 2/41 | 43.00 ± 12.00 | 43.00 ± 11.00 | 35 | 35 |
| Konstantinos et.al., 2016 | Greece | NA | NA | NA | NA | NA | 44 | 43 |
| Tadic et.al., 2016^*^ | Italy | 2.5 | 0/39 | 0/39 | 42.00 ± 12.00 | 43.00 ± 12.00 | 39 | 39 |
| Tadic et.al., 2016^†^ | Italy | 2.5 | 0/45 | 0/24 | 43.00 ± 12.00 | 43.00 ± 12.00 | 45 | 24 |
| Liu et.al., 2019 | China | 3.5 | 6/39 | 6/28 | 40.10 ± 12.40 | 38.37 ± 12.50 | 47 | 34 |
| Wang et.al., 2019 | China | 2.0-4.0 | 10/23 | 12/23 | 39.00 ± 11.60 | 32.4 ± 14.00 | 33 | 35 |
| Duzen et.al., 2020 | Turkey | 2.0-4.0 | 13/27 | 11/29 | 32.90 ± 11.70 | 33.30 ± 11.90 | 40 | 40 |
| Zhou et.al., 2020 | China | 1.5-4.6 | 8/39 | 6/28 | 40.150 ± 12.31 | 38.77 ± 12.50 | 47 | 34 |

Abbreviations: MHz, Mega Hertz; SD, standard deviation; NA, not available.

^*^ study published by J Ultrasound Med, ^†^ study published by Scandinavian Cardiovascular Journal.

**Supplementary Table 2. Quality assessment of included studies by Newcastle-Ottawa Scale^*^**

| **Studies included** | **Overall quality score** |
| --- | --- |
| Jiang et.al., 2015 | 7 |
| Fu et.al., 2015 | 7 |
| Tadic et.al., 2015 | 6 |
| Konstantinos et.al., 2016 | 6 |
| Tadic et.al., 2016a | 6 |
| Tadic et.al., 2016b | 7 |
| Liu et.al., 2019 | 6 |
| Wang et.al., 2019 | 7 |
| Duzen et.al., 2020 | 7 |
| Zhou et.al., 2020 | 7 |

^*^ The study quality was assessed by Newcastle Ottawa Quality assessment scale for cross-sectional studies or cohort studies. This scale awards a maximum of 9 points to each study.

**Supplementary Table 3. Measurements of two-dimensional echocardiographic parameters in patient and control groups**

|  | **Fu et.al., 2015** | **Tadic et.al., 2015** | **Konstantinos et.al., 2016** | **Tadic et.al., 2016** | **Tadic et.al., 2016** | **Liu et.al., 2019** | **Duzen et.al., 2020** | **Duzen et.al., 2020** | **Zhou et.al., 2020** |
| --- | --- | --- | --- | --- | --- | --- | --- | --- | --- |
| **Controls** |  |  |  |  |  |  |  |  |  |
| LVEF (%) | 65.03±3.02 | NA | 63.18±4.20 | 65.00±3.00 | 65.00±3.00 | 68.88±4.22 | 62.00±4.00 | 62.00±4.00 | 66.43±3.91 |
| IVST (mm) | NA | NA | 8.70±1.20 | 8.80±1.10 | 8.80±1.00 | NA | 9.00±0.20 | 9.00±0.20 | NA |
| LVEDD (mm) | 47.00±1.64 | 46.30±3.00 | 50.50±3.20 | 46.00±3.00 | 46.20±3.40 | 42.59±3.04 | 44.00±4.00 | 44.00±4.00 | NA |
| LVEDS (mm) | 26.87± 1.71 | 29.10±3.40 | 31.70±4.60 | 29.50±3.60 | 30.30±3.10 |  | 33.00±1.75 | 33.00±1.75 | NA |
| LV mass index (g/m^2^) | NA | 88.10±16.40 | 80.67±9.12 | 38.60±4.30 | NA | NA | 80.35±1.95 | 80.35±1.95 | NA |
| **Patients** |  |  |  |  |  |  |  |  |  |
| LVEF (%) | 67.89±3.02 | NA | 57.43±6.40 | 64.00±4.00 | 65.00±3.00 | 63.70±18.95 | 62.5±5.00 | 62.50±5.00 | 65.62±7.02 |
| IVST (mm) | NA | NA | NA | NA | NA | NA | NA | NA | NA |
| LVEDD (mm) | NA | NA | 9.23±2.40 | 9.30±0.90 | 9.10±1.00 | NA | NA | NA | NA |
| LVEDS (mm) | 46.41±1.92 | 47.00± 2.90 | 53.5±8.14 | 47.00±3.00 | 47.10±3.60 | 43.75±3.68 | 48.00±6.25 | 47.00±4.00 | NA |
| LV mass index (g/m^2^) | 8.61±0.57 | NA | NA | NA | NA | NA | NA | NA | NA |
| LVEF (%) | NA | 100.80±19.70 | 95.28±11.87 | 43.80±4.80 | NA | NA | 98.5±7.70 | 90.70±10.65 | NA |

Abbreviations: LVEF, left ventricular ejection fraction; IVST, interventricular septal thickness; LVEDD, left ventricular end-diastolic diameter; LVEDS, left ventricular end systolic diameter; NA, not available.

**Supplementary Table 4. Measurements of three-dimensional echocardiographic parameters in patient group and the control group (part A)**

| **LV-left ventricle** | **Jiang et.al., 2015** | **Fu et.al., 2015** | **Wang et.al., 2019** | **Tadic et.al., 2015** | **Wang et.al., 2019** |
| --- | --- | --- | --- | --- | --- |
| **Controls** |  |  |  |  |  |
| LVEF (%) | 62.79±7.69 | 67.89±3.02 | 65.27±2.34 | 62.00±3.00 | 65.27±2.34 |
| EDMASS | 110.45±15.67 | NA | NA | NA | NA |
| ESMASS | 111.65±14.79 | NA | NA | NA | NA |
| RR | NA | 840.21±139.89 | NA | NA | NA |
| **Cases** |  |  |  |  |  |
| LVEF (%) | 63.47±8.21 | 54.93±1.02 | 64.64±2.60 | 62.00±4.00 | 64.64±2.6 |
| EDMASS | 112.32±14.65 | NA | NA | NA | NA |
| ESMASS | 113.12±15.43 | NA | NA | NA | NA |
| RR | NA | 699.95±142.27 | NA | NA | NA |

Abbreviations: LVEF, LV ejection fraction; IVST, interventricular septal thickness; LVEDD, left ventricular end-diastolic diameter; LVEDS, left ventricular end systolic diameter; NA, not available.

**Supplementary Table 5. Measurements of three-dimensional echocardiographic parameters in patient group and the control group (part B)**

| **LV-left ventricle** | **Jiang et.al., 2015** | **Fu et.al., 2015** | **Wang et.al., 2019** | **Tadic et.al., 2015** | **Wang et.al., 2019** |
| --- | --- | --- | --- | --- | --- |
| **Controls** |  |  |  |  |  |
| Global longitudinal strain (%) | -18.65±2.93 | -18.52±4.77 | NA | NA | NA |
| Global circumferential strain (%) | -18.54±3.07 | -25.88±2.12 | NA | NA | NA |
| Global area strain (%) | 32.56±4.12 | NA | NA | NA | NA |
| Global radial strain (%) | 48.51±8.47 | NA | NA | NA | NA |
| **Cases** |  |  |  |  |  |
| Global longitudinal strain (%) | -27.11±3.02 | -20.47±3.08 | NA | NA | NA |
| Global circumferential strain (%) | -24.87±3.22 | -28.98±3.00 | NA | NA | NA |
| Global area strain (%) | 36.13±4.25 | NA | NA | NA | NA |
| Global radial strain (%) | 53.67±6.12 | NA | NA | NA | NA |

Abbreviations: LVEF, LV ejection fraction;NA, not available.

**Supplementary Table 6. Measurements of three-dimensional echocardiographic parameters in patient group and the control group (part C)**

| **LV-left ventricle** | **Jiang et.al., 2015** | **Fu et.al., 2015** | **Wang et.al., 2019** | **Tadic et.al., 2015** | **Wang et.al., 2019** |
| --- | --- | --- | --- | --- | --- |
| **Controls** |  |  |  |  |  |
| LVEDD (mm) | NA | NA | NA | NA | 4.46±0.36 |
| LVEDS (mm) | NA | NA | NA | NA | 2.51±0.31 |
| LVEDV (mm) | NA | 98.13±19.94 | NA | 119.00±20.00 | NA |
| LVESV (mm) | NA | 44.19±8.88 | NA | 44.00±14.00 | NA |
| **Cases** |  |  |  |  |  |
| LVEDD (mm) | NA | NA | NA | NA | 4.61±0.35 |
| LVEDS (mm) | NA | NA | NA | NA | 2.61±0.28 |
| LVEDV (mm) | NA | 107.07±18.87 | NA | 131.00±22.00 | NA |
| LVESV (mm) | NA | 44.85±8.78 | NA | 50.00±16.00 | NA |

Abbreviations: LVEDD, left ventricular end-diastolic diameter; LVEDS, left ventricular end systolic diameter; NA, not available.

**Supplementary Table 7. Measurements of three-dimensional echocardiographic parameters in patient group and the control group (part D)**

| **LV-left ventricle** | **Jiang et.al., 2015** | **Fu et.al., 2015** | **Wang et.al., 2019** | **Tadic et.al., 2015** | **Wang et.al., 2019** |
| --- | --- | --- | --- | --- | --- |
| **Controls** |  |  |  |  |  |
| Stroke volume | NA | NA | NA | 75.00±17.00 | NA |
| Cardiac output (l/min) | NA | NA | NA | 5.30±0.80 | NA |
| LV mass index (g/m2) | NA | NA | NA | 80.10±13.20 | NA |
| Sphericity index | NA | NA | NA | 0.41±0.05 | NA |
| LV-TW | NA | 10.52±7.21 | NA | NA | NA |
| LV-TOR | NA | 1.34±0.95 | NA | NA | NA |
| LV-PROTA | NA | 4.82±4.18 | NA | NA | NA |
| **Cases** |  |  |  |  |  |
| Stroke volume | NA | NA | NA | 81.00±18.00 | NA |
| Cardiac output (l/min) | NA | NA | NA | 6.00±0.90 | NA |
| LV mass index (g/m2) | NA | NA | NA | 89.60±15.10 | NA |
| Sphericity index | NA | NA | NA | 0.39±0.05 | NA |
| LV-TW | NA | 14.76±6.12 | NA | NA | NA |
| LV-TOR | NA | 1.89±0.77 | NA | NA | NA |
| LV-PROTA | NA | 6.55±4.20 | NA | NA | NA |

Abbreviations: LVEF, LV ejection fraction; IVST, interventricular septal thickness; LVEDD, left ventricular end-diastolic diameter; LVEDS, left ventricular end systolic diameter; NA, not available.

**Supplementary Table 8. Publication bias of summarized outcomes**

| **Outcomes** | **Begg (*P* value)** | **Egger (*P* value)** |
| --- | --- | --- |
| Summary of two-dimensional echocardiographic parameters for left ventricular control group | 0.22 | 0.94 |
| Summary of two-dimensional echocardiographic parameters for left ventricular patient group | 0.84 | 0.74 |
| Summary of three-dimensional echocardiographic parameters for left ventricular control group | 0.34 | 0.37 |
| Summary of three-dimensional echocardiographic parameters for left ventricular patient group | 0.60 | 0.49 |

Abbreviations: MRI, magnetic resonance imaging; NA, not available.
